# Transcriptional Regulation and Its Misregulation in Human Diseases

**DOI:** 10.3390/ijms24108640

**Published:** 2023-05-12

**Authors:** Amelia Casamassimi, Alfredo Ciccodicola, Monica Rienzo

**Affiliations:** 1 Department of Precision Medicine, University of Campania “Luigi Vanvitelli”, Via L. De Crecchio, 80138 Naples, Italy; amelia.casamassimi@unicampania.it; 2 Institute of Genetics and Biophysics “Adriano Buzzati-Traverso”, CNR, Via P. Castellino 111, 80131 Naples, Italy; 3 Department of Science and Technology, University of Naples “Parthenope”, 80143 Naples, Italy; 4 Department of Environmental, Biological, and Pharmaceutical Sciences and Technologies, University of Campania “Luigi Vanvitelli”, 81100 Caserta, Italy; monica.rienzo@unicampania.it

Transcriptional regulation is a critical biological process that allows the cell or an organism to respond to a variety of intra- and extracellular signals, to define cell identity during development, to maintain it throughout its lifetime, and to coordinate cellular activity. This control involves multiple temporal and functional steps, as well as innumerable molecules, including transcription factors, cofactors, and chromatin regulators. It is well known that many human disorders are characterized by global transcriptional dysregulation, since most of the signaling pathways ultimately target transcription machinery. Indeed, many syndromes and genetic and complex diseases, including cancer, autoimmunity, neurological and developmental disorders, and metabolic and cardiovascular diseases, can be caused by mutations/alterations in regulatory sequences, transcription factors, splicing regulators, cofactors, chromatin regulators, ncRNAs, and other components of transcription apparatus. It is worth noting that advances in our understanding of molecules and mechanisms involved in the transcriptional circuitry and apparatus lead to new insights into the pathogenetic mechanisms of various human diseases and disorders. Thus, this Special Issue is focused on molecular genetics and genomics studies, exploring the effects of transcriptional misregulation on human diseases [1,2].

In this Special Issue, a total of 18 excellent and interesting papers [3,4,5,6,7,8,9,10,11,12,13,14,15,16,17,18,19,20], consisting of 13 original research studies [3,4,5,6,10,11,12,14,15,16,18,19,20], as well as five reviews, are published [7,8,9,13,17]. They cover many subjects of transcriptional regulation, including studies on cis-regulatory elements, transcription factors (TF), chromatin regulators, and ncRNAs. As expected, a substantial number of transcriptome studies and computational analyses are also included in this issue.

Significantly, all the published papers have provided novel insights on the knowledge of human pathophysiological mechanisms, having the purpose of proposing novel biomarkers and/or therapeutic targets for the diagnosis and treatment of many diseases, especially cancer. In the field of TFs, two papers by Nagel et al. [3,4] are focused on the class of homeobox genes, encoding developmental factors containing a homeodomain with three helices, which interact with DNA, cofactors, and chromatin, thus allowing gene-regulating activities essential for the control of basic cell and tissue differentiation decisions. In accordance with their normal functions, these genes, when deregulated, contribute to carcinogenesis along with hematopoietic malignancies. In one of the manuscripts [3], they established the so-called myeloid TALE-code, representing a TALE homeobox gene expression pattern in normal myelopoiesis. The class of TALE homeobox genes comprises specific homeodomain factors that share a three-amino-acid residue loop extension (abbreviated as TALE) between helix 1 and helix 2. These transcription factors control basic developmental decisions. The same authors had previously constructed the lymphoid TALE-code that codifies expression patterns of all active TALE class homeobox genes in early hematopoiesis and lymphopoiesis, thus extending the TALE code to the entire hematopoietic system. Collectively, data showed expression patterns for eleven TALE homeobox genes and highlighted the exclusive expression of IRX1 in megakaryocyte-erythroid progenitors, suggesting that this TALE class member is involved in a specific myeloid differentiation route. Interestingly, the analysis of public transcription profiles from acute myeloid leukemia (AML) patients revealed aberrant expression of IRX1, IRX3, and IRX5, indicating an oncogenic role for these TALE homeobox genes when deregulated in AML [3].

In the second work [4], Nagel and colleagues investigated the subclass of NKL homeobox genes that also function in normal development and are often deregulated in hematopoietic malignancies; indeed, a previous systematic analysis revealed 18 deregulated NKL homeobox genes in AML, underlining the relevance of these developmental oncogenes in driving this cancer type. In this newly published study, the authors also identified aberrantly activated NKL genes, NKX2-3 and NKX2-4, in cell lines derived from two different AML subtypes, where they deregulate target genes involved in megakaryocytic and erythroid differentiation, thus providing the molecular basis to the classification of specific AML subtypes [4].

Another TF, which is involved in tumor progression and metastasis is Yin-Yang transcription factor 1 (YY1); indeed, it also overexpressed in different cancers, including leukemia. Antonio-Andres, G et al. [5] observed that the expression of *YY1* in patients with pediatric acute lymphoblastic leukemia (ALL) positively correlates with *HIF1A* transcription. Besides, their findings clearly indicate, for the first time, that *YY1* is transcriptionally regulated by HIF-1α and suggest that both *HIF1A* and *YY1* transcription factors could be possible therapeutic targets and/or biomarkers of ALL [5].

A further regulatory mechanism that may play a role in tumor development and progression involves two additional TFs, Forkhead Box Protein P3 (*FOXP3*) and activating transcription factor 3 (*ATF3*) [6]. FOXP3 has an essential and critical role in autoimmunity, cancer development, and Treg development, with hundreds of target genes already identified in both cancer cells and Treg cells. Additionally, *FOXP3* is a recognized breast and prostate tumor suppressor gene from the X chromosome, acting as a transcriptional repressor for several oncogenes. Using several human cell lines, Chiung-Min Wang et al. [6] assessed the function of *FOXP3* in the transcriptional activity of *ATF3*, which binds several promoters of key regulatory proteins that determine cell fate, circadian signaling, and homeostasis, and it is rapidly induced by many pathophysiological signals and is essential in cellular stress response. Overall, their findings suggest that *FOXP3*, through FOX protein response element, functions as a novel repressor of *ATF3* and that phosphorylation at Y342 plays a critical role for *FOXP3* transcriptional activity [6].

Besides, three reviews of this Special Issue are mainly focused on TFs. The first one discusses the regulation of *SNAI1* (Snail Family Transcriptional Repressor 1), a zinc finger transcription factor, which acts as a master regulator of epithelial–mesenchymal transition (EMT). Noteworthy, *SNAI1* is involved in the formation of cancer metastases by epigenetic regulation and post-translational modifications [7]. The Waku and Kobayashi [8] review describes the pathophysiological aspects of the biomolecular pathways regulated by NRF3 (NFE2L3; NFE2-like BZIP Transcription Factor 3), a transcription factor belonging to the cap‘n’collar (CNC)-based leucine zipper family and functioning through proteasome regulation. The NRF3 factor and its regulated axes are involved in cancer cell growth and have anti-obesity potential, thus suggesting a possible role in the development of obesity-induced cancer [8]. Finally, Rai V. et al. critically summarized the studies performed on the regeneration of sensory hair cells (HCs) in adult mammalian cochleas to elucidate the molecular pathways, and particularly transcription factors, involved in the regeneration of cochlear HCs, which aids in proposing a biological approach for better therapeutics to treat hearing loss and to restore hearing [9].

In the last years, the old annotation of protein-coding genes, based on the presence of an open reading frame (ORF) with minimal lengths for translated proteins, has significantly changed. Indeed, the recent literature indicates that the proteome is more complex than previously estimated, since RNAs previously considered noncoding, such as long noncoding RNAs (lncRNAs) and circular RNAs, are instead translated into functional small proteins [10]. In an interesting study of this Special Issue, the authors utilized transcriptome and polysome profiling to identify novel micropeptides that originate from lncRNAs that are expressed exclusively in hepatocellular carcinoma (HCC) cells, but not in the liver or other normal tissues. Specifically, they found three HCC-specific lncRNAs, containing at least one ORF longer than 50 amino acid (aa) and enriched in the polysome fraction. Besides, through a peptide specific antibody, they characterized one lncRNA candidate, NONHSAT013026.2/Linc013026-68AA, which is translated into a 68 aa micropeptide. This small protein is mainly localized at the perinuclear region and is mainly expressed in moderately—but not well-differentiated—HCC cells, and it plays a role in cell proliferation, suggesting that it could be used as an HCC-specific target molecule. This finding is noteworthy, since it represents an important advance in the study of the previously overlooked “dark proteome” and its role in human pathologies, particularly in cancer [10].

Another emerging field, especially in cancer, is represented by chimeric RNAs, which are transcripts consisting of exons from different parental genes. They can be produced by several mechanisms mostly involving chromosomal rearrangements; besides, they can also be generated by intergenic splicing, cis-splicing from two same-strand adjacent genes, and trans-splicing from two separate RNA transcripts [11]. Although these events were initially considered rare, human transcriptome profiles have revealed that a huge amount of chimeric RNAs develop from intergenic splicing and can be also detected in normal tissues, thus contributing to transcriptomic complexity. An interesting study has analyzed the genetic structure and biological roles of *CLEC12A-MIR223HG*, a novel chimeric transcript produced through trans-splicing by the fusion of the cell surface receptor CLEC12A (C-Type Lectin Domain Family 12 Member A) and the miRNA-223 host gene (*MIR223HG*), first identified through transcriptome profiling of chronic myeloid leukemia (CML) patients. Unexpectedly, *CLEC12A-MIR223HG* was detected not only in CML, but also in a variety of normal tissues and cell lines as pro-monocytic cells resistant to chemotherapy or during monocyte-to-macrophage differentiation [11]. Transcriptional activation of CLEC12A increased *CLEC12A-MIR223HG* expression. This chimeric RNA also translates into a chimeric protein, which largely resembles CLEC12A, but contains a modified C-type lectin domain, altering key disulphide bonds. Consequently, differences in post-translational modifications, cellular localization, and protein–protein interactions occur. These findings not only support a possible involvement of *CLEC12A-MIR223HG* in the regulation of CLEC12A function, but they could also provide a roadmap to study the other uncharacterized chimeric RNAs that are continuously recognized by RNA-Seq analyses [11].

In the last decades, next generation sequencing (NGS) strategies have been greatly applied in a huge number of cancer studies with different purposes. Among the NGS applications, several DNA barcode-based parallel reporter methods have been implemented for the screening of regulatory risk sites. Among them, the dinucleotide reporter system (DiR)-seq screening system was developed to investigate the gene regulatory effect from the risk single nucleotide polymorphisms (SNPs) that have a modest impact. In their paper, Ren and coworkers [12] applied the DiR system in prostate cancer cells (22Rv1) to screen the regulatory risk SNPs, leading to transcriptional misregulation, and they identified 32 regulatory SNPs that exhibited different regulatory activities with two alleles. Among them, fourteen SNPs exhibited decreased expression levels for the risk alleles, whereas eighteen SNPs showed increased expression. Particularly, they discovered that the rs684232 T allele altered chromatin binding of transcription factor FOXA1 on the DNA region and led to aberrant gene expression of *VPS53*, *FAM57A*, and *GEMIN4*, which are often upregulated in prostate cancer patients. Thus, these findings provide novel insights to further elucidate the basis mechanism of the functional prostate cancer risk SNPs [12]. 

An important role in the mechanisms of transcriptional regulation is also played by transposable elements, repetitive genetic sequences with the ability (sometimes lost during evolution) to transpose elsewhere in the genome. A class of these elements is represented by the endogenous retroviruses (ERVs), which represent about 8% of the sequences present in the human genome. An interesting overview of this Special Issue [13] describes the mechanisms underlying their transcriptional regulation. During the evolution of the human genome, the accumulation of mutations, insertions, deletions, and/or truncations has rendered these elements inactive. However, it is increasingly evident that, under the influence of genetic and epigenetic mechanisms, they can be involved in some physiological and pathological conditions; examples are their function in embryonic development, or, even more importantly, their reactivation in the development of human diseases, such as cancer and neurodegenerative disorders [13]. Besides, a remarkable paper [14] shows that transcriptional alterations observed in X-linked dystonia–parkinsonism (XDP) are caused by the insertion of a SINE-VNTR-Alu (SVA) retrotransposon in an intron of the *TAF1* (TATA-Box Binding Protein Associated Factor 1) gene, encoding for the largest subunit of TFIID; as an interesting effect, increased levels of the *TAF1* intron retention transcript *TAF1-32i* can be found in XDP cells, as compared to healthy controls. Overall, the results of this study provide further evidence that transposable elements affect gene expression and suggest that a mechanism of splicing alteration occurs in XDP patients, probably caused by binding sites for transcription factors and splicing regulators present within this retrotransposon and that need to be exactly proved through additional experiments [14].

Currently, transcriptome profiling is one of the most utilized approaches to investigate human diseases at the molecular level [2]. Here, Kim and colleagues [15] compare the transcriptomic profiles, extracted from the NCBI Gene Expression Omnibus (GEO) database, of brown adipose tissue (BAT) of young and elderly subjects in response to thermogenic stimuli. Interestingly, they observe that aging does not cause transcriptional changes in thermogenic genes, but it upregulates several pathways related to the immune response and downregulates metabolic pathways. Furthermore, they note that acute severe cold exposure (CE) upregulates several pathways related to protein folding, whereas chronic mild CE upregulates metabolic pathways, mostly related to carbohydrate metabolism [15].

Furthermore, in the study of Suojalehto et al. [16], transcriptome profiling was assessed to investigate whether a distinct clinical subtype of adult-onset asthma could be related to damp and moldy buildings, which are symptoms of idiopathic environmental intolerance, thus identifying potential molecular similarities with this disease. To this purpose, fifty female adult-onset asthma patients were categorized based on their exposure to building dampness and molds and other clinical parameters (inflammation, cytokine profile, etc.), together with gene signatures of nasal biopsies and peripheral blood mononuclear cells. Overall, the results of this study revealed a greater degree of similarity between idiopathic environmental intolerance and dampness related asthma than between the same patients and those with asthma not associated to dampness and mold [16]. Transcriptome analysis revealed well defined pathological mechanisms for asthma without exposure to dampness, but not dampness-and-mold-related asthma patients. Besides, a distinct molecular pathological profile in nasal and blood immune cells of idiopathic environmental intolerance subjects was found, including several differentially expressed genes (DEGs) that were also detected in dampness-and-mold-related asthma samples, thereby suggesting idiopathic environmental intolerance-type mechanisms [16].

It is well known that eukaryotic transcription is a complex, biological, and stepwise process, ranging from initiation, elongation, and termination to the process of pre-mRNA with 5′-end capping, splicing, 3′-end cleavage, and polyadenylation; subsequently, the mature mRNA is exported from the nucleus to the cytoplasm to undergo protein translation, whereas the aberrantly processed pre-mRNAs and mRNAs are removed via the RNA surveillance system. All these steps are also interconnected with each other, and with chromatin accessibility and additional epigenetic mechanisms [1]. Of note, alteration of any step may constitute the basis of a disease. For instance, Park HS. et al. [17] illustrate recent findings on the role of the nuclear mRNAs export in cellular aging and age-related neurodegenerative disorders. Indeed, it is now established that there is a close relationship between transcription and aging; however, the role of the nuclear mRNAs export in these issues is still poorly characterized. Of note, disruption of the regulation of factors mediating mRNA export from the nucleus (namely, TREX, TREX-2, and nuclear pore complex) has been shown to result in the accumulation of aberrant nuclear mRNAs, with the consequent alteration of normal lifespan and the development of neurodegenerative diseases [17].

Concerning epigenetics, an interesting paper focused on obesity without metabolic complications, a phenotype defined as metabolically healthy obesity (MHO), which can progress to an unhealthy state known as metabolically unhealthy obesity (MUO), although a relevant percentage of MHO individuals are likely to maintain their status over time [18]. The authors aimed to analyze the long-term evolution of DNA methylation patterns in a subset of MHO subjects in order to search for epigenetic markers that could predict the progression of MHO to MUO. As a result, twenty-six CpG sites were significantly differentially methylated, both at baseline and after eleven years of follow-up. Two potential biomarkers of the transition to an unhealthy state were identified: specifically, higher methylation in cg20707527 (*ZFPM2*) and lower methylation in cg11445109 (*CYP2E1*) could impact the stability of a healthy phenotype in obesity [18].

Finally, the study of transcriptional regulation is also useful to elucidate the pathogenetic mechanisms of human pathogens. Bacterial sensing of environmental signals has an essential role in modulating virulence and bacterium–host interactions. Generally, bacteria utilize the two-component system (TCS) method, such as QseEF, to control gene expression in response to rapid environmental changes. Of note, many recognized mechanisms function through the post-transcriptional control of small non-coding RNAs (sRNAs), and their identification is rapidly increasing in number and variety in the context of bacteria regulatory functions [19]. Interestingly, in a study in the present Special Issue, the authors identified the QseEF homologue of *Proteus mirabilis*, an Important pathogen of the urinary tract, principally in patients with indwelling urinary catheters, and they found it is involved in the modulation of swarming motility through the sRNA *GlmY*. This is the first study investigating a pathway mediated by a two-component system through a sRNA as an underlying pathogenetic mechanism of *Proteus mirabilis* swarming migration, during which expression of several virulence genes is increased. Since it is assumed that *P. mirabilis* swarming up catheters is primed to infect the urinary tract, clarifying the swarming mechanisms could provide new approaches in the development of intervention strategies and facilitate the discovery of novel therapeutics [19].

Again, in the field of human pathogens, Braun et al. [20] focused on the molecular diagnosis of the anthrax pathogen *Bacillus anthracis*, which is challenging because its identification is complicated by the close relationship with other bacteria of the group species. The authors have designed and validated an ultrasensitive detection method that can be run as a real-time PCR with solely DNA as a template or as a RT-real-time version using both cellular nucleic acid pools (DNA and RNA) as a template without requirement of DNase treatment. This assay was found to be highly species specific, yielding no false positives, and it was highly sensitive targeting a unique single nucleotide polymorphism within a variable number of loci of the multi-copy 16S rRNA gene and related transcripts. With the high abundance of 16S rRNA moieties in cells, it is expected to facilitate the detection of *B. anthracis* by PCR. While standard PCR assays are well established for the identification of *B. anthracis* from pure culture, the exceptional sensitivity of the new 16S rRNA-based test might excel in clinical and public health laboratories when detection of minute residues of the pathogen is required [20].

In conclusion, we hope the readers enjoy this Special Issue of IJMS and the effort to present the current advances and promising results in the field of transcriptional regulation and its involvement in all the relevant biological processes and in pathophysiology.

## Data Availability

Not applicable.
